# Microbiological Quality of Fresh Nopal Juice

**DOI:** 10.3390/microorganisms4040046

**Published:** 2016-12-10

**Authors:** Ana María Hernández-Anguiano, Patricia Landa-Salgado, Carlos Alberto Eslava-Campos, Mateo Vargas-Hernández, Jitendra Patel

**Affiliations:** 1 Phytopathology, Graduate College, Carr. Mexico-Texcoco km 36.5, Montecillo, Texcoco 56230, Mexico; patylanda@gmail.com; 2 Departamento de Salud Pública, Facultad de Medicina, National Autonomous University of Mexico (UNAM), University Avenue No. 3000, Ciudad Universitaria, Coyoacan, Mexico City 04510, Mexico; eslava@unam.mx; 3 Unidad de Hemato Oncología e Investigación, Hospital Infantil de México “Federico Gómez”/División de Investigación, Facultad de Medicina UNAM. Dr. Márquez 162, Col. De los Doctores, Cd. de Mexico 06720, Mexico; 4 Soil Science, Autonomous University of Chapingo, Km 38.5 Carretera Mexico-Texcoco, Chapingo 56230, Mexico; vargas_mateo@hotmail.com; 5 U.S. Department of Agriculture, Agricultural Research Service, Beltsville, MD 20705, USA; Jitu.Patel@ARS.USDA.GOV

**Keywords:** cactus, *Opuntia-ficus* indica, nopal juice, *Citrobacter*, *E. coli*, total coliforms, *Salmonella*

## Abstract

The consumption of fresh nopal cactus juice is widely popular among health-conscious consumers in Mexico. The juice is prepared from fresh cladodes that have only been rinsed with tap water and are not subjected to a pasteurization or terminal bacterial reduction process. The aim of this study was to evaluate the microbial quality of commercially available fresh juices (*n* = 162) made with nopal in Texcoco, State of Mexico, during the summer and spring season. Standard microbiological methods, the PCR technique and the serological method were used for isolation and identification of bacteria. All samples contained total coliforms and 91% were positive for *Escherichia coli*. Although total coliforms and *E. coli* were detected throughout the study, their populations were significantly lower (*p* < 0.05) in winter and spring, respectively. *Citrobacter youngae* was found in 20% of the samples, an unidentified species of *Citrobacter* in 10%, *C. freundii* and *Proteus mirabilis* in 3%, and *Salmonella* Javiana in 1%. The presence of these microorganisms, especially *Salmonella*, in the nopal juices is unacceptable due to its health significance. The information generated in this study is relevant for human health risk assessment associated with the consumption of unpasteurized nopal juices and potential interventions to minimize pathogen contamination.

## 1. Introduction

Nopal cactus (*Opuntia ficus*-indica) (L.) Mill. (Cactaceae) is a native species of economic and social importance in Mexico. The tender stems (10–15 cm) of nopal, commonly known as “nopales”, “nopalitos” or “cladodes” (flattened stems), are used as main ingredients in a wide variety of cooked (soups and sauces) or raw (salads and juices) food products due to their high content of dietetic fiber, minerals, vitamins and antioxidants [1].

The consumption of fresh nopal juice, alone or combined with other fresh fruits and vegetables, is widely popular among health-conscious consumers due to its purported health benefits. In addition to its nutritive value, the juice made from nopal is also consumed for its medicinal properties such as antiulcer and hypoglycemic activity [2,3], skin healing properties [4], and of its ability to reduce blood serum cholesterol [1]. Therefore, it is consumed by people, including those with type 2 diabetes mellitus, either in natural form, blended with water or mixed with other products such as pineapple, orange or grapefruit [5].

In the central zone of Mexico, commercial vendors in the buildings and local street stands offer fresh nopal juice for immediate consumption or to be consumed later. These vendors prepare juice from fresh nopal cladodes that have only been rinsed with tap water and the juice not subjected to a pasteurization or terminal bacterial reduction process. Fresh cattle manure is commonly used for soil fertility in traditional nopal agricultural farming practices. Hernandez and others [6] have reported the persistence of *Salmonella* in fresh nopal cladodes harvested at the farms following traditional farming practices. Potential contamination of nopal cladodes by *Salmonella* and other enteropathogens via soil at the farm level can cause outbreaks of foodborne illnesses as unpasteurized nopal juice is consumed. In the absence of terminal treatment for removing pathogens from nopal cladodes, the fresh juices made with nopal can be a vehicle for pathogens such as *Salmonella* and others [7,8]. Previous studies have reported the presence of bacterial pathogens in unpasteurized beverages prepared with fresh fruits and vegetables [9,10,11,12]. The main aim of this study was to evaluate the microbiological quality of fresh juices made with vegetable nopal sold at two commercial stands in the central zone of Texcoco, State of Mexico.

The information generated in this study is relevant for developing new interventions for bacterial reduction in fresh juice and for risk assessment for human health represented by the consumption of fresh unpasteurized nopal juice.

## 2. Materials and Methods

### 2.1. Sampling of Fresh Juice

Freshly prepared nopal juice samples (*n* = 162) were collected every month during June 2009 to May 2010 from commercial stands in the central zone of Texcoco, State of Mexico. Two stands were chosen based on a daily fresh nopal juice availability, potable water availability for juice preparation, and hygienic practices. One of the stands (stand A) located inside the building had potable tap water available whereas the other stand (stand B) is on the local street did not have tap water and had potential for airborne contamination in outside environment. Usually stand A served more than 10 types of fresh nopal juices, and stand B, just one type, during the sampling year. Types of fresh nopal juices analyzed in this study are shown in Table 1. A 500 mL fresh nopal juices samples, prepared from chopped nopal cladodes, without spines, and blended with fresh squeezing citric juice and/or a mix of fresh fruits and vegetables, were collected at both stands (138 samples from Stand A and 24 from stand B) in Styrofoam container sanitized with alcohol. Usually during the summer, fall and winter season, stand A served the first 12 types of fresh nopal juices indicated in Table 1 but not the following seven type of juices. In contrast, during the spring season of 2010 this stand served the last seven type of juices but not the first 12 types. At each sampling point, nopal cladode juice was prepared with natural water to determine reference microbial quality of cladode. The juice samples were transported on ice, maintained on ice or in refrigeration in the laboratory and processed within 2 h of sampling time.

### 2.2. Microbiological Analysis

Prior to the microbiological analysis, the pH of each sample was determined with a potentiometer (Hanna Instruments, Sarmeola di Rubano, Italy). Because most of the juice nopal types were dense, samples for microbiological analysis were taken by weight (g) instead of volume (mL). To determine total coliforms using the plate count technique [13], 25 g of each juice sample was mixed with 225 mL of 0.1% buffered peptone water (BPW, Becton Dickinson, Cuautitlan Izcally, Mexico) in a sterile bottle and manually mixed for 15 s. Serially diluted juice suspensions were spread-plated on MacConkey Agar (MA, Becton Dickinson, Cuautitlán Izcally, Mexico). After 24 h incubation at 37 °C, lactose fermenting red colonies were counted as total coliforms. Presumptive pink or red *E. coli* colonies on MacConkey agar plates were identified following comparative pink or red colonies of reference strain *E. coli* 042 on MacConkey agar.

The isolation of presumptive *Salmonella* was carried out in non-selective pre-enrichment medium and selective enrichment medium [14]. For the non-selective pre-enrichment, 10 g of each sample was mixed with 90 mL tryptic soy broth (TSB, Bioxon) in sterile bottle and incubated overnight at 37 °C. Following incubation, 1 mL each was transferred to 9 mL of tetrathionate base broth (TB, Becton Dickinson), and 9 mL of Vassiliadis-Rappaport broth (VRB, Becton Dickinson) and incubated at 37 °C for 18–24 h. A loopful of selectively enriched samples were streaked on Hektoen enteric agar (HEA, Bioxon) and on bright green agar (BGA, Bioxon) plates and incubated at 37 °C for 24 h. Following incubation, select *Salmonella* colonies that are green or blue-green, with or without a black center, or completely black colonies [15] in HEA were transferred in Luria-glycerol (20%, v/v) at −20 °C. These *Salmonella* colonies were confirmed with biochemical tests using Vitek automated system (BioMérieux, Hazelwood, MO, USA) and the polymerase chain reaction (PCR) technique. From each positive juice sample, at least two isolates were retained for further confirmation and characterization.

### 2.3. Biochemical Identification and Characterization

Eighty-eight presumptive *Salmonella* isolates preserved in Luria-glycerol (20%) were reactivated in TSB and grown in HEA for further characterization. Presumptive *Salmonella* isolates were evaluated for indole production, methyl red, Voges-Proskauer test and citrate production, mobility analysis, hydrogen sulphate production, deamination and decarboxylation of lysine, sugar production and fermentation of sugars (glucose, lactose and saccarose) [16].

Cryopreserved *Salmonella* isolates were reactivated in blood gelose agar for confirmation of biochemical identity with the Vitek system (BioMerieux, Hazelwood, MO, USA). The reactivated cultures were grown in soy tryptone agar at 37 °C for 24 h, subsequently diluted in 0.4% saline solution and then adjusted to 1 OD in the McFarland scale for the automatic card completion of Gram Negative (GN) cards. After 24 h at 37 °C, Vitek system identified isolates based on biochemical results.

### 2.4. Serological and Molecular Confirmation

One of the presumptive *Salmonella* isolates and phenotypically identified by Vitek system as *Salmonella* was sent to the Bacteriology Laboratory of the Department of Public Health of the Department of Medicine of UNAM to determine the serogroup by somatic and flagellar agglutination method [17]. For the amplification of the InvA gene of *Salmonella* [18], the primers Sal-3 and Sal-4 were used with PCR Core System 1 (Promega, Madison, WI, USA). Cellular lysate samples (raw DNA) of *Salmonella* Javiana strain, raw DNA of three strains of the undefined genus between *C. youngae* and *C. freundii*, and raw DNA of a *C. freundii* strain, all of them isolate from nopal juice samples, were analyzed with PCR method [18]. Lysates of *S. enterica* serotype Typhimurium ATCC23564 and *E. coli* 042 were used as positive and negative controls, respectively.

### 2.5. Statistical Analysis

The pH values and microbiological results of samples ran in triplicate were analyzed with SAS program (Windows 9.4). Analyses of variance due to seasonal effect, type of juice, and their interactions were conducted using PROC GLM and multiple comparison of means based on the honest significant difference of Tukey at 5% significance level (*p* = 0.05).

## 3. Results and Discussion

The 162 samples of fresh juice made from fresh nopal had an average pH of 4.6, with a range of 4.1 to 5.1 depending on the type of juice. When the pH values were analyzed, significant differences (α = 0.05) were found among the pH of the juices (Table 2).

**Figure 1:**
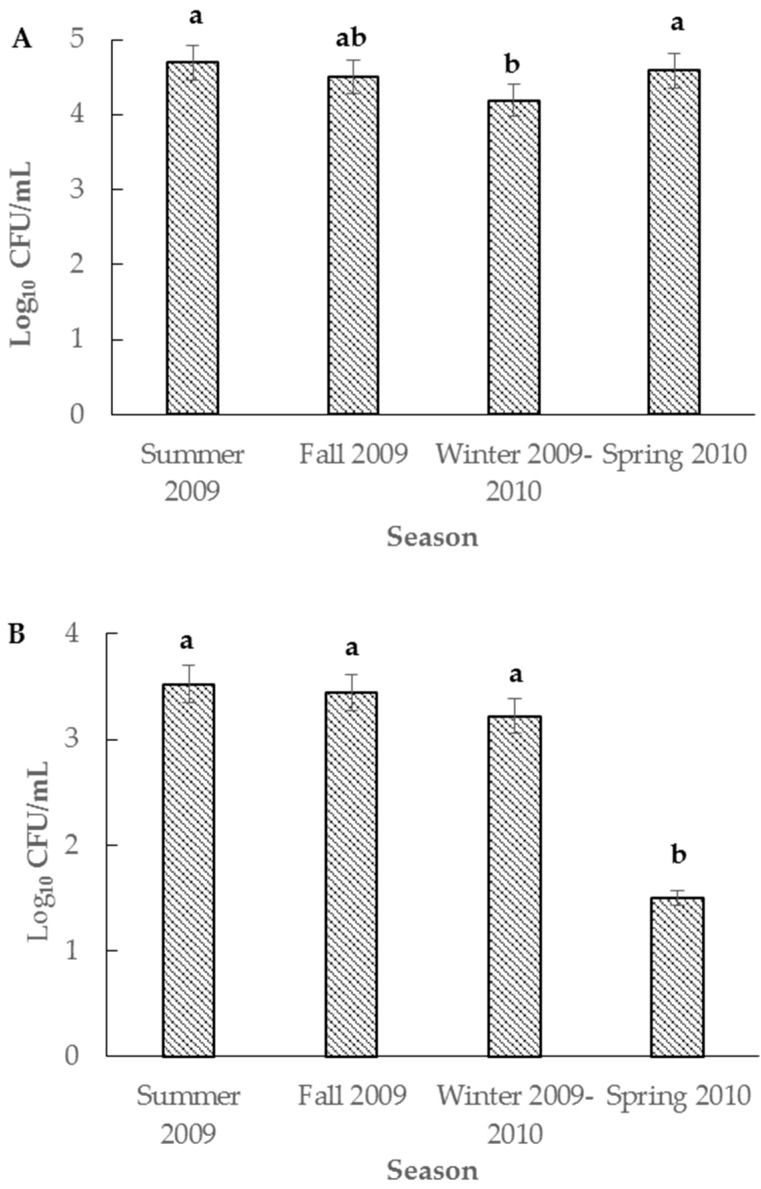
Total coliforms (**A**) and presumptive *E. coli* (**B**) population in nopal juices collected during June 2009 and May 2010. In total, 162 samples were analyzed: summer, 42 samples; fall, 45 samples; winter, 45 samples; and spring, 30 samples. Means with the same letter are not statistically different.

Total coliforms were detected in all juice samples (100%) with an average value of 4.5 Log CFU/mL. The total coliforms varied significantly with the type of juice and season of sample collection. The nopal, celery, lemon, parsley and carrot juice had the highest total coliforms (5.6 Log CFU/mL); the nopal, garlic, lemon, parsley and pineapple juice had the lowest coliforms (3.3 Log CFU/mL, Table 2). While the total coliforms varied in different types of juices, the average coliforms in samples collected in winter (4.1 Log CFU/mL) were significantly lower than those samples obtained in the summer season (Figure 1A). Presumptive *E. coli* colonies were detected throughout the year, but their count was significantly lower (*p* < 0.05) (1.6 Log CFU/mL) during the spring-2010 season (Figure 1B). Presumptive *E. coli* was detected in 148 of 162 samples (91%) with an average of 3.0 Log CFU/mL. Most of the *E. coli*- negative nopal juice samples were collected during April and May of 2010. Similar to the total coliforms, *E. coli* populations were also significantly influenced by the type of juice and season of sample collection. The samples of nopal, celery, lemon, parsley and carrot juice had the highest presumptive *E. coli* populations (4.8 Log CFU/mL) whereas the least presumptive *E. coli* (0.9 Log CFU/mL) populations were detected in nopal, olive oil, sugar beet, flax, papaya, honey and orange juice samples (Table 2).

According to the Norm NOM-127-SSA1 standards [19], potable tap water should be free from total coliforms and *E. coli*. Although total coliforms do not necessarily represent a risk for the health of consumers, the higher coliforms in fresh nopal juice indicate the poor microbial quality of nopal, potable water, or an inadequate hygienic process during their preparation. Similarly, the correlation between generic *E. coli* and enteric pathogens is unknown; however, the recent Food Safety Modernization Act (FSMA) by the US-FDA requires monitoring irrigation water for generic *E. coli* to control enteric pathogens on fresh produce [20]. It is possible that the extent of contamination could be dependent on how the juice was prepared [21]. The collection of potable water at each juice-sampling schedule and subsequent water analysis could have helped in identifying the role of water in the contamination of fresh juice. Further, the poor hygiene practices, such as washing hands or equipment used during preparation, could also be involved in the contamination of the juices. Our results on the lower recovery of coliforms during cold seasons are in agreement with the results reported by Mollie and Groisman [22] who observed the seasonal effects of *Salmonella* and *E. coli* survival in fresh vegetables following manure application.

**Figure 2:**
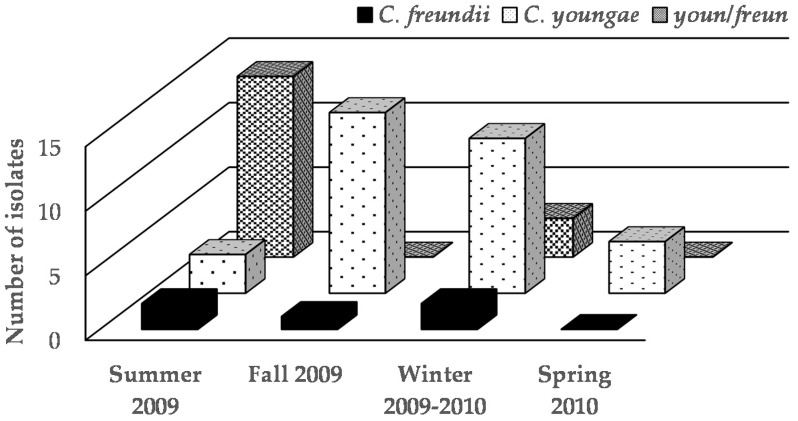
*Citrobacter* species isolated from fresh nopal juices collected during June 2009 and May 2010; youn/freun corresponds to strains of indefinite species between *C. youngae* and *C. freundii*.

In our study, 62 presumptive colonies of *Salmonella* were isolated from 29 samples using HEA agar. The biochemical characterization identified 57 of these isolates as *Citrobacter freundii* and five as *Proteus mirabilis*. When analyzed by the automated system, the identity of the strains of *Citrobacter* resulted as *C. youngae* (33 strains), *C. freundii* (five strains), unidentified species between *C. youngae* and *C. freundii* (17 strains), *Salmonella* (one strain) and an unidentified strain (Table 3). It should be mentioned that *P. mirabilis* and the *Salmonella* strain were isolated from samples of nopal natural water juice collected at the local street (stand B) in the summer and fall, respectively, of 2009. *Citrobacter* populations were influenced by the type of juice and season of sample collection (Table 2, Figure 2).

The genera of *Proteus* and *Citrobacter* are related to *Salmonella*, all pertaining to the family Enterobacteriaceae. In general, *Citrobacter* and *Salmonella* are difficult to distinguish by biochemical analysis [23]; even in their serological characterization, crossed reactions can be identified in the analysis of the somatic antigens O [24]. We used a larger number of biochemical tests and a commercial identification system to precisely differentiate these bacteria as suggested by O’Hara et al. [25].

**Figure 3:**
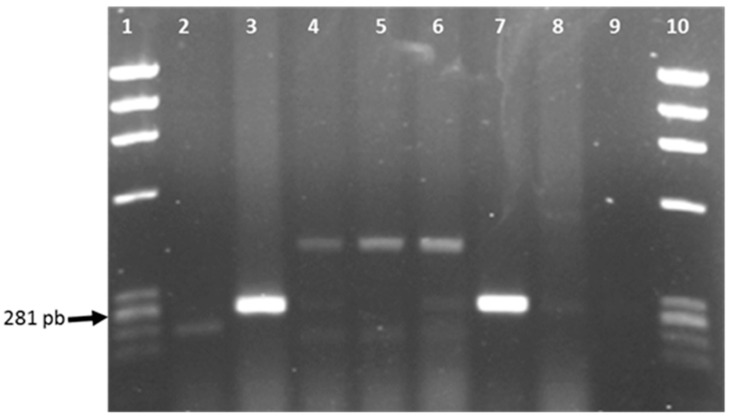
PCR amplification products with Sal 3 and Sal 4 of cell lysates. Lanes: 1 and 10, Marker DNA/Hae; Lanes 2, 4 and 5, strains of indeterminate species of *C. youngae* and *C. freundii*; Lane 3, strain of *S*. Javiana; Lane 6, strain of *C. freundii*; Lane 7, *S*. Typhimurium ATCC23564; Lane 8, *E. coli* 042; Lane 9, water.

The *Salmonella* strain was characterized as the *S. enterica* serotype Javiana (*S*. Javiana) through somatic and flagellar agglutination; the PCR reaction generated a positive result of amplification with a band (275 bp) similar to that obtained with the positive control (*S*. Typhimurium ATCC23564) (Figure 3). Although the presence of *S*. Javiana has been reported previously in vegetable nopal [6], the potential source of *Salmonella* contamination was obscure in this study. In addition to water quality and hygienic practices, the presence of flies at both stands could have also contributed to *Salmonella* contamination in the fresh juice. Contamination of fresh nopal juice with *Proteus*, *Citrobacter* and *Salmonella* represents a serious threat to healthy and immuno-compromised consumers who consume it for health benefits. Although *P. mirabilis* and *C. freundii* are considered opportunist pathogens [16], they can cause diseases in humans such as neonatal meningitis and urinary tract infections, respectively, in immuno-compromised people [26,27]. It is well known that the consumption of fresh unpasteurized juices, even with a low pH, represents a risk for human health. *Salmonella* was the causative agent of infections in 64% of the foodborne outbreaks associated with fresh juice [12]. Castillo et al. [9] reported the presence of *Salmonella* in 20% of fresh orange juice samples collected at local street stands in Guadalajara, Mexico. It should be pointed out that *S*. Javiana is a highly virulent serotype, which has been implicated in various outbreaks associated with the consumption of contaminated fruits, vegetables and other products [28].

The consumption of fresh fruit and vegetable juices is widely popular with consumers because of their purported health benefits. In general, fruit juice consumers have the idea that fresh fruit juices are of minimal concern for microbial safety because of the very low probability of finding human pathogens in these products due to their low pH and high acidity. However, several juice-related outbreaks of salmonellosis have been registered during the last two decades [29,30,31]. This indicates that *Salmonella* has the ability to tolerate acidic conditions for survival and causes disease upon consumption of the juice.

## 4. Conclusions

The microbiological quality of the fresh juices prepared with vegetable nopal, blended with fresh squeezed citrus juice and/or a mix of fresh fruits and vegetables, in commercial establishments in Texcoco, State of Mexico, is unacceptable based on the persistence of enteric microorganisms in the juices. These enteropathogens can survive and cause serious infections in healthy and immuno-compromised people. The monitoring of the microbial quality of the fresh products and water used in juice preparation, the potential terminal treatment to reduce microbial populations in fresh juice, personal hygiene, and sanitation practices at vendor stands are important control points to be considered for reducing the risks to human health. The information generated in this study is relevant for developing new interventions for bacterial reduction in fresh juice and for risk assessment for human health represented by the consumption of fresh unpasteurized nopal juice.

## Figures and Tables

**Table 1:** Characteristics of fresh nopal juices collected monthly from June 2009 to May 2010.

Stand	Juice Composition	Recommendation *
**In building (A)**		
A	Nopal, natural water	
A	Nopal, celery, orange, lemon, pineapple, and purslane	Cholesterol
A	Nopal, oats, orange and apple	Cholesterol
A	Nopal, broccoli, cabbage, asparagus and grapefruit	Glucose
A	Nopal, aloe vera, grapefruit and xoconostle	Diabetes
A	Nopal, garlic, lemon, parsley and pineapple	Reumas
A	Nopal, celery, sugar beet and pineapple	Slim down
A	Nopal, celery, lemon, parsley and carrot	Clean stomach
A	Nopal, squash, orange, cucumber and pineapple	Kidneys
A	Nopal, natural water, aloe vera and xoconostle	Matrix and cysts
A	Nopal, celery, parsley and pineapple	Diet
A	Nopal, prunes, flaxseed, papaya and orange	Constipation
A	Nopal, natural water, parsley and sugar beet	High triglycerides
A	Nopal, celery, honey, orange and pineapple	Diet
A	Nopal, oats, apple, honey and orange	Cholesterol 1
A	Nopal, alfalfa, lemon, honey, orange and pineapple	Cholesterol 2
A	Nopal, natural water, aloe vera and xoconostle	Diabetes
A	Nopal, olive oil, sugar beet, flax, papaya, honey and orange	Laxative
A	Nopal, aloe vera, grape fruit and xoconostle	Cysts
A	Nopal, prunes, flaxseed, honey, papaya and orange	Constipation
**In street booth (B)**		
B	Nopal, natural water	
B	Nopal, celery, orange, parsley and pineapple	Diet

* Health recommendation were posted by vendors and not necessarily evaluated by authors.

**Table 2:** Incidence of coliforms and number of enteropathogen isolates in nopal juices.

Stand-Juice Composition	pH	TC Log_10_	*E. coli* Log_10_	*Citrobacter*	*Proteus*	*Salmonella*
**In building**						
Nopal, natural water	4.9 abcd	4.3 cdef	2.7 bc	7		
Nopal, celery, orange, lemon, pineapple, and purslane	4.5 defg	4.8 abcd	3.6 ab	1		
Nopal, oats, orange and apple	4.5 cdefg	4.4 cdef	3.1 bc			
Nopal, broccoli, cabbage, asparagus and grapefruit	4.4 defg	4.2 def	3.3 bc			
Nopal, aloe vera, grapefruit and xoconostle	4.3 efg	4.2 cdef	3.4 b			
Nopal, garlic, lemon, parsley and pineapple	4.3 efg	3.3 g	2.1 cd			
Nopal, celery, sugar beet and pineapple	4.5 cdefg	4.9 abcd	3.8 ab	7		
Nopal, celery, lemon, parsley and carrot	5.1 ab	5.6 a	4.8 a	10		
Nopal, squash, orange, cucumber and pineapple	4.4 defg	4.7 bcde	3.4 b			
Nopal, natural water, aloe vera and xoconostle	4.6 bcdefg	4.4 cdef	3.1 bc	18		
Nopal, celery, parsley and pineapple	4.5 cdefg	5.1 abc	3.8 ab	2		
Nopal, prunes, flaxseed, papaya and orange	4.7 bcdef	4.3 cdef	3.2 bc	2		
Nopal, natural water, parsley and sugar beet	4.7 bcdefg	4.7 abcd	3.4 bc			
Nopal, celery, honey, orange and pineapple	4.1 g	5.3 ab	1.3 de			
Nopal, oats, apple, honey and orange	4.5 cdefg	4.6 bcde	1.2 de			
Nopal, alfalfa, lemon, honey, orange and pineapple	4.2 fg	4.9 abcd	1.0 e			
Nopal, natural water, aloe vera and xoconostle	4.3 efg	4.1 defg	1.0 de			
Nopal, olive oil, sugar beet, flax, papaya, honey, orange	5.0 abc	4.8 bcd	0.9 e			
Nopal, aloe vera, grape fruit and xoconostle	4.6 bcdefg	4.7 bcde	1.3 g	3		
Nopal, prunes, flaxseed, honey, papaya and orange	4.8 abcd	3.9 efg	1.0 e			
**In street booth**						
Nopal, natural water	4.9 abcd	4.5 cdef	3.1 bc	5	5	1
Nopal, celery, orange, parsley and pineapple	4.5 defg	3.7 fg	2.89 bc			

Means with same letter are not statistically different.

**Table 3:** Biochemical characterization of presumptive *Salmonella* strains isolated from nopal juices.

Month	Stand-Juice	Number of Strains	Characterization	
			Biochemical	VITEK 2
June	A-Nopal, natural water, aloe vera and xoconostle	4	*C. freundii* (4) ^¥^	*C. youngae/C. freundii* (3) *C. youngae* (1)
July	A-Nopal, celery, lemon, parsley and carrot	2	*C. freundii* (2)	*C. youngae/C. freundii* (2)
	A-Nopal, natural water, aloe vera and xoconostle	3	*C. freundii* (3)	Unidentified (1) *C. youngae/C. freundii* (2)
August	A-Nopal, celery, orange, lemon, pineapple, and purslane	1	*C. freundii* (1)	*C. freundii* (1)
	A-Nopal, celery, sugar beet and pineapple	5	*C. freundii* (5)	*C. youngae/C. freundii* (4)
				*C. freundii* (1)
	A-Nopal, celery, lemon, parsley and carrot	1	*C. freundii* (1)	*C. youngae* (1)
	A-Nopal, prunes, flaxseed, papaya and orange	2	*C. freundii* (2)	*C. youngae/C. freundii* (1)
				*C. youngae* (1)
	B-Nopal, natural water	3	*C. freundii* (3)	*Salmonella* (1)
				*C. youngae/C. freundii* (2)
September	A-Nopal, celery, lemon, parsley and carrot	7	*C. freundii* (7)	*C. youngae* (7)
	A-Nopal, natural water, aloe vera and xoconostle	1	*C. freundii* (1)	*C. freundii* (1)
October	A-Nopal, celery, sugar beet and pineapple	2	*C. freundii* (2)	*C. youngae* (2)
	A-Nopal, celery, lemon, parsley and carrot	2	*C. freundii* (2)	*C. youngae* (2)
	B-Nopal, natural water	1	*C. freundii* (1)	*C. youngae* (1)
November	A-Nopal, celery, parsley and pineapple	2	*C. freundii* (2)	*C. youngae* (2)
December	A-Nopal, natural water	5	*C. freundii* (5)	*C. youngae* (5)
	A-Nopal, natural water, aloe vera and xoconostle	5	*C. freundii* (5)	*C. freundii* (2) *C. youngae/C. freundii* (3)
	B-Nopal, natural water	2	*C. freundii* (2)	*C. youngae* (2)
January	A-Nopal, natural water, aloe vera and xoconostle	2	*C. freundii* (2)	*C. youngae* (2)
February	A-Nopal, natural water	2	*C. freundii* (2)	*C. youngae* (2)
March	A-Nopal, celery, lemon, parsley and carrot	1	*C. freundii* (1)	*C. youngae* (1)
April	A-Nopal, natural water, aloe vera and xoconostle	1	*C. freundii* (1)	*C. youngae* (1)
May	A-Nopal, aloe vera, grape fruit and xoconostle	3	*C. freundii* (3)	*C. youngae* (3)

^¥^ In parenthesis number of strains.
